# Predictive value of folate receptor-positive circulating tumor cells in postoperative progressive disease of non-small cell lung cancer patients

**DOI:** 10.1007/s00432-026-06554-1

**Published:** 2026-06-28

**Authors:** Wenjing Zhou, Mengyuan Song, Jiajia Song, Litin You, Yi Zhu, Hao Chen, Juan Zhou

**Affiliations:** 1 Department of Laboratory Medicine, West China Hospital, Sichuan University, Chengdu, 610041 China; 2 Clinical Laboratory Medicine Research Center, West China Hospital, Sichuan University, Chengdu, 610041 China; 3 Department of Laboratory Medicine, General Healthcare Chengfei Hospital, University, Chengdu, 610041 China

**Keywords:** Folate receptor-positve circulating tumor cells, Non-small cell lung cancer, Progressive disease

## Abstract

**Background:**

Postoperative recurrence and metastasis constitute critical obstacles for resected non-small cell lung cancer (NSCLC) patients, and reliable predictive biomarkers are still scarce. This study aimed to evaluate whether folate receptor-positive CTC (FR^+^CTC) predicts postoperative progressive disease (PD) in stage I–IIIA NSCLC and to explore noninvasive biomarkers for high-risk patients.

**Method:**

We retrospectively enrolled stage I–IIIA NSCLC patients undergoing radical resection from 2018 to 2021. Perioperative blood samples were collected for FR^+^CTC detection. PD-related risk factors were analyzed to establish a postoperative prognostic model.

**Result:**

In total, 171 NSCLC patients were enrolled. Stage III disease had higher preoperative FR⁺CTC positivity than stage I–II (69.39% vs. 52.46%, *P* = 0.043). Male gender, smoke, squamous histology and stage III correlated with elevated postoperative FR⁺CTC positivity (all *P* < 0.05). Among 152 patients for survival analysis, 36 developed 36‑month PD. Multivariate Cox confirmed postoperative FR⁺CTC positivity (HR = 2.25, *P* = 0.018) and stage III (HR = 3.43, *P* = 0.005) as independent PD predictors. FR⁺CTC conversion from preoperative negative to postoperative positive conferred the highest PD risk and shortened PFS.

**Conclusion:**

Postoperative FR^+^CTC positive and TNM stage III were independent risk factors for 36 months PD in NSCLC patients after radical resection. Postoperative FR⁺CTC positivity correlated with shortened PFS and effectively predicted postoperative PD. Conversion from preoperative FR⁺CTC negativity to postoperative positivity conferred the highest progression risk. Perioperative FR⁺CTC detection and dynamic monitoring are promising liquid biopsy biomarkers for postoperative progression prediction.

## Introduction

Worldwide, lung cancer remains the malignancy with the highest incidence and mortality rates (Siegel et al. 2024). Non-small cell lung cancer (NSCLC) is the most common histological subtype, accounting for approximately 87% of all cases (Debieuvre et al. 2022). NSCLC is highly aggressive, with an overall 5-year survival rate of approximately 25%.By contrast, the 5-year survival rate declines markedly to lower than 15% among patients who develop metastasis or recurrence (Boeschen et al 2023, Aggarwal et al 2023).Radical resection remains the first-line treatment choice for patients with resectable early-stage NSCLC (Chinese Medical Association guideline 2025). However, following radical resection, some patients still suffer from progressive disease(PD), recurrence or metastasis, the overall 3-year recurrence rate ranges from approximately 14.9 to 36.8%, with about 80% of PD occurring within the first 2 years post-surgery and leading to treatment failure (Potter et al. 2023, Renaud et al. 2025, Altorki et al. 2023).NSCLC patients exhibit marked heterogeneity in histology type, molecular characteristics, and driver genes (Scalera et al. 2021), resulting in considerable variability in the risk of PD after resection among different patients. Currently, there is a lack of reliable biomarkers to help identify NSCLC patients at high risk of postoperative PD.

As a important non-invasive molecular marker in liquid biopsy, circulating tumor cells (CTCs) have demonstrated significant clinical value in the early diagnosis, prognosis assessment, treatment response monitoring, minimal residual disease (MRD) detection, and drug resistance prediction of lung cancer (Normanno et al. 2025). CTCs originate from primary tumor lesions and disseminate through the bloodstream or lymphatic system. Their presence is considered a critical predictor of tumor micrometastasis and distant metastasis (Borea et al. 2025). Studies have shown that detection of high levels of CTCs before or after surgery is significantly associated with increased risk of tumor metastasis and early postoperative recurrence (Pascual et al. 2022, Volovetsky et al. 2025, Chinniah et al. 2019). The folate receptor (FR) is highly expressed in various epithelial-derived malignant tumors, especially in lung cancer cells, compared with normal tissues, FR expression is markedly up-regulated in tumor tissues of approximately 75.7% of NSCLC patients (O’Shannessy et al. 2012), making FR an ideal marker for CTC detection in lung cancer. Previous studies have indicated that the level of folate receptor-positive circulating tumor cell (FR^+^CTC) in NSCLC patients is significantly correlated with tumor aggressiveness (Lv et al. 2022). However, research on the association between FR^+^CTC and postoperative PD in Chinese NSCLC patients undergoing radical resection remains limited, even fewer has investigated the correlation between dynamic changes in FR^+^CTC before and after surgery and PD risk.

Here we enrolled patients with stage I-IIIA NSCLC, collected their clinical data, and measured FR^+^CTC levels before and short after surgery to explore their potential as biomarkers for predicting the risk of postoperative PD in NSCLC patients.

## Materials and methods

### Study subjects

We enrolled patients with NSCLC patients who underwent radical surgery at West China Hospital, Sichuan University from January 2018 to May 2021. Follow-up continued until May 2023. Inclusion criteria were: ① Treatment-naive patients diagnosed according to established guidelines (Chinese Medical Association guideline 2025); ② Age ≥ 18 years; ③ Underwent radical resection; ④pathological TNM (pTNM) stage I-IIIA. Exclusion criteria were: ① History of other malignant tumors; ② Severe active infection/communicable diseases; ③ Pulmonary diseases such as emphysema or active pulmonary tuberculosis; ④ History of multiple chemotherapy cycles or extensive radiotherapy. This study was approved by the Biomedical Ethics Committee of West China Hospital, Sichuan University (No. 1045,2019).

### FR^+^CTC detection

#### Specimen collection

Peripheral blood samples (4 mL each) were collected into EDTA-anticoagulated tubes from 1 to 7 days before surgery. Postoperative specimens were obtained on postoperative days 3 to 7. Samples were stored at 4 °C and FR^+^CTC detection was completed within 24 h after collection.

#### FR + CTC detection

Preoperative and postoperative FR^+^CTC were performed using CytoploRare® kit provided by GenoSaber Biotech Co. Ltd. (Shanghai, China) (Yu et al. 2014). FR^+^CTC ≥ 8.70 FU/3 mL was defined as “FR^+^CTC positivity” (FR^+^CTC[+]), and FR^+^CTC < 8.70 FU/3 mL was defined as “FR^+^CTC negativity” (FR^+^CTC[-]) (Chen et al 2015, Lou et al 2013).

#### Patient clinical data collection and follow-up

Baseline data, clinical characteristics, adjuvant therapy information, and survival outcomes were collected.Baseline characteristics included gender (male/female), age, histological type, pTNM stage, and smoking status (smoke was defined as continuous or cumulative tobacco use for ≥ 6 months during one’s lifetime). Follow-up procedures and criteria for progressive disease (recurrence/metastasis) were defined according to the “Chinese Expert Consensus on Postoperative Follow-up for Non-Small Cell Lung Cancer (2020 Edition)” (Lunxu et al. 2021).

### Statistical analysis

Statistical analyses were performed using SPSS software (version 26.0). Categorical variables were compared using the chi‑square test. Survival curve analysis was performed using the Kaplan–Meier method with log-rank test.The Cox proportional hazards regression model was used for univariable and multivariable analyses. R software (version 4.2.1) was used to generate forest plots based on the Cox model, and to plot Kaplan–Meier curves. A *P*-value < 0.05 was considered statistically significant.

## Result

### Clinical characteristics of the NSCLC patients

A total of 171 patients with NSCLC were enrolled, with a median age of 59 years. Of these patients, 78 (45.61%) were female and 93 (54.39%) were male.97 patients (56.72%) were smokers. Regarding histological type, adenocarcinoma (ADC) was the most common, accounting for 128 patients (74.85%), while squamous cell carcinoma (SCC) was diagnosed in 43 patients (25.15%). According to the TNM staging system, 122 patients (71.34%) were classified as stage I-II, and 49 patients (28.65%) were stage III, as detailed in Table 1.

**Table 1:** Clinical characteristics of the NSCLC patients

Characteristics		Number of patients (%)	
Age (years)	<60	87(50.88)	
	≥ 60	84(49.12)	
Gender	Female	78(45.61)	
	Male	93(54.39)	
Smoking status	Yes	97(56.73)	
	No	74(43.27)	
Histological type	ADC	128(74.85)	
	SCC	43(25.15)	
TNM stage	I + II	122(71.34)	
	III	49(28.65)	

### Correlation between patient FR^+^CTC and clinical characteristics

#### Correlation between preoperative FR^+^CTC and patient clinical characteristics

Among the 171 patients, 98 (57.30%) were preoperative FR^+^CTC[+]. Patients were divided into two groups based on their preoperative FR^+^CTC results. Comparison of clinical characteristics between the two groups revealed that TNM stage III patients had a significantly higher preoperative FR^+^CTC positivity rate compared to stage I-II patients (69.39% vs. 52.46%, *P* = 0.043). The risk of positive preoperative FR^+^CTC was 2.05 higher in stage III patients to stage I + II patients (OR = 2.05, 95% CI: 1.01–4.15, *P* = 0.043). No statistically significant differences were observed between the two groups in age, gender, histological type, or smoke (*P* > 0.05), as shown in Table 2.

**Table 2:** Preoperative FR^+^CTC of NSCLC patients

Variables		FR^+^CTC[-] *n*(%)	FR^+^CTC[+] *n*(%)	OR(95%CI)	P*
Age	≧ 60	35(41.67)	49(58.33)	1.09(0.59–1.99)	0.79
	< 60	38(43.68)	49(56.32)		
Gender	Male	37(39.78)	56(60.22)	1.30(0.71–2.39)	0.402
	Female	36(46.15)	42(53.85)		
Smoking status	Yes	28(37.84)	46(62.16)	1.42(0.77–2.63)	0.263
	No	45(46.39)	52(53.61)		
Histological type	SCC	17(39.53)	26(60.47)	1.19(0.59–2.41)	0.629
	ADC	56(43.75)	72(56.25)		
TNM stage	III	15(30.61)	34(69.39)	2.05(1.01–4.15)	**0.043**
	I-II	58(47.54)	64(52.46)		

*Multivariate analysis *P* value. Bold values indicate statistical significance ( *P *< 0.05).

#### Correlation between postoperative FR^+^CTC and patient clinical characteristics

To investigate the correlation between postoperative FR⁺CTC status and clinical characteristics, patients were stratified into a postoperative FR^+^CTC[-] group and a postoperative FR^+^CTC[+] group based on the postoperative FR^+^CTC results.The results demonstrated that male sex (38.71% vs. 24.36%), smoke (40.54% vs. 25.77%), SCC subtype (53.49% vs. 25.00%), and TNM stage III (44.90% vs. 27.05%) were significantly associated with higher postoperative FR⁺CTC positivity rates (*P* = 0.045, 0.041, < 0.001, and 0.024, respectively). No significant difference was observed with respect to age (*P* > 0.05), as presented in Table 3.

**Table 3:** Postoperative FR^+^CTC of NSCLC patients

Variables		FR^+^CTC[-] *n*(%)	FR^+^CTC[+] *n*(%)	OR(95%CI)	*P*
Age	≧ 60	53(63.10)	31(36.90)	1.54(0.81–2.93)	0.192
	< 60	63(72.41)	24(27.59)		
Gender	Male	57(61.29)	36(38.71)	1.96(1.01–3.81)	**0.045**
	Female	59(75.64)	19(24.36)		
Smoking status	Yes	44(59.46)	30(40.54)	1.96(1.03–3.76)	**0.041**
	No	72(74.23)	25(25.77)		
Histological type	SCC	20(46.51)	23(53.49)	3.45(1.68–7.09)	**< 0.001**
	ADC	96(75.00)	32(25.00)		
TNM stage	III	27(55.10)	22(44.90)	2.20(1.10–4.38)	**0.024**
	I + II	89(72.95)	33(27.05)		

Bold values indicate statistical significance (P< 0.05).

### Risk factors for postoperative PD within 36 months in NSCLC patients

To investigate risk factors for early postoperative PD, we conducted subsequent analysis on 152 patients who had a follow-up duration exceeding 36 months. Among these patients, 52 (34.21%) patients received postoperative adjuvant therapy,36 (23.68%) experienced PD. Of these 36 PD cases, 12 occurred within the first 12 months after surgery, 12 occurred between 13 and 24 months, and 12 occurred between 25 and 36 months postoperative.

Univariable analyses were performed to evaluate the associations between progressive disease (PD) and clinicopathological variables.We observed that the PD rate was significantly higher in the following groups: male sex (32.93% vs. 12.86%), smoke (39.39% vs. 11.63%), SCC subtype (46.15% vs. 15.93%), TNM stage III (53.33% vs. 11.21%), administration of adjuvant therapy (42.31% vs. 14.00%), and postoperative FR^+^CTC[+] (40.00% vs. 15.69%).All differences were statistically significant (*P* = 0.004, < 0.001, < 0.001, < 0.001, < 0.001, and < 0.001, respectively), as shown in Table 4.

**Table 4:** Univariable analysis of risk factors for postoperative PD within 36 months in NSCLC patients

Variables		no-PD *n*(%)	PDn(%)	HR(95%CI)	*P*
Age (years)	≧ 60	54(75.00)	18(25.00)	1.15(0.54–2.43)	0.717
	< 60	62(77.50)	18(22.50)		
Gender	Male	55(67.03)	27(32.93)	3.32(1.44–7.69)	**0.004**
	Female	61(87.14)	9(12.86)		
Smoking status	Yes	40(60.60)	26(39.39)	4.94(2.17–11.26)	**< 0.001**
	No	76(88.37)	10(11.63)		
Histological type	SCC	21(53.85)	18(46.15)	4.52(2.02–10.13)	**< 0.001**
	ADC	95(84.07)	18(15.93)		
TNM stage	III	21(46.67)	24(53.33)	9.05(3.91–20.93)	**< 0.001**
	I–II	95(88.79)	12(11.21)		
Adjuvant therapy	Yes	30(57.69)	22(42.31)	4.50(2.05–9.91)	**< 0.001**
	No	86(86.00)	14(14.00)		
Preoperative FR^+^CTC	Positive	66(72.52)	25(27.47)	1.72(0.78–3.83)	0.180
	Negative	50(81.97)	11(18.03)		
Postoperative FR^+^CTC	Positive	30(60.00)	20(40.00)	3.58(1.64–7.80)	**< 0.001**
	Negative	86(84.31)	16(15.69)		

Bold values indicate statistical significance ( P < 0.05).

**Fig. 1:**
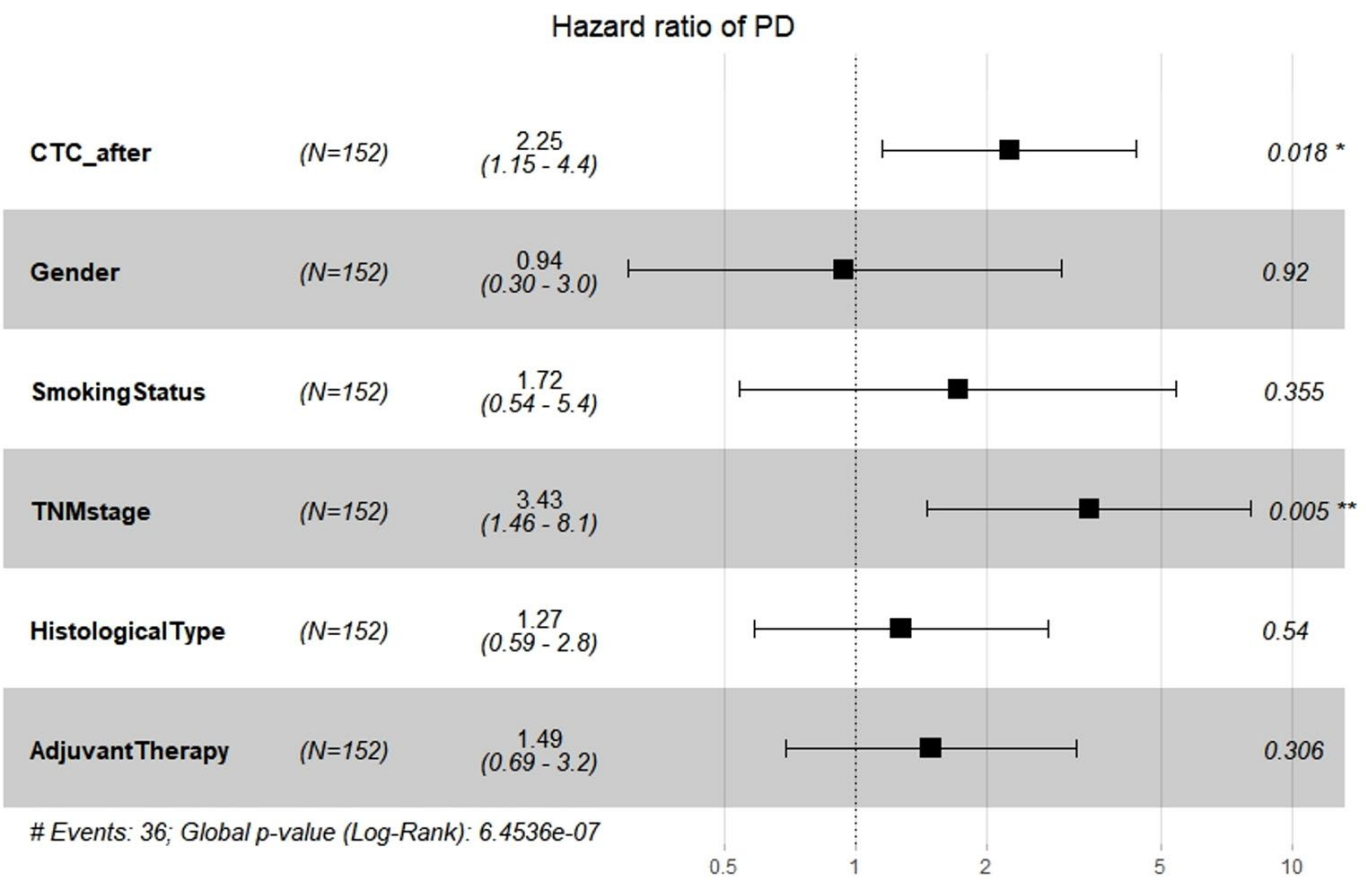
Forest plot showing multivariate Cox proportional hazards for postoperative PD within 36 months in NSCLC patients

After identifying potential risk factors inunivariable analysis, we further performed multivariate Cox proportional hazards regression to screen for independent predictors of PD within 36 months after surgery. Six variables with significant differences in univariable analysis were entered into the model: gender, smoking status, histological type, pTNM stage, adjuvant therapy, and postoperative FR⁺CTC status. The results are visualized in the Cox regression forest plot (Fig. 1). As shown in Fig. 1, postoperative FR^+^CTC[+] (HR = 2.25, 95% CI: 1.16–4.40, *P* = 0.018) and TNM stage III (HR = 3.43, 95% CI: 1.46–8.15, *P* ≡ 0.005) were identified as independent risk factors for postoperative PD. Gender, smoking status, histological type, and adjuvant therapy showed no statistically significant associations with postoperative PD( *P* = 0.920, *P* = 0.355, *P* = 0.540, *P* = 0.306).The model demonstrated good discriminatory performance, with a global likelihood ratio χ² *P* = 6.45 × 10⁻7 and a concordance index (C‑index) of 0.80, based on 36 events among 152 patients.

### Kaplan–Meier curve of PFS in NSCLC patients within 36 months

Kaplan-Meier curves were generated to assess the progression-free survival (PFS) within 36 months postoperative in these 152 NSCLC patients .

**Fig. 2:**
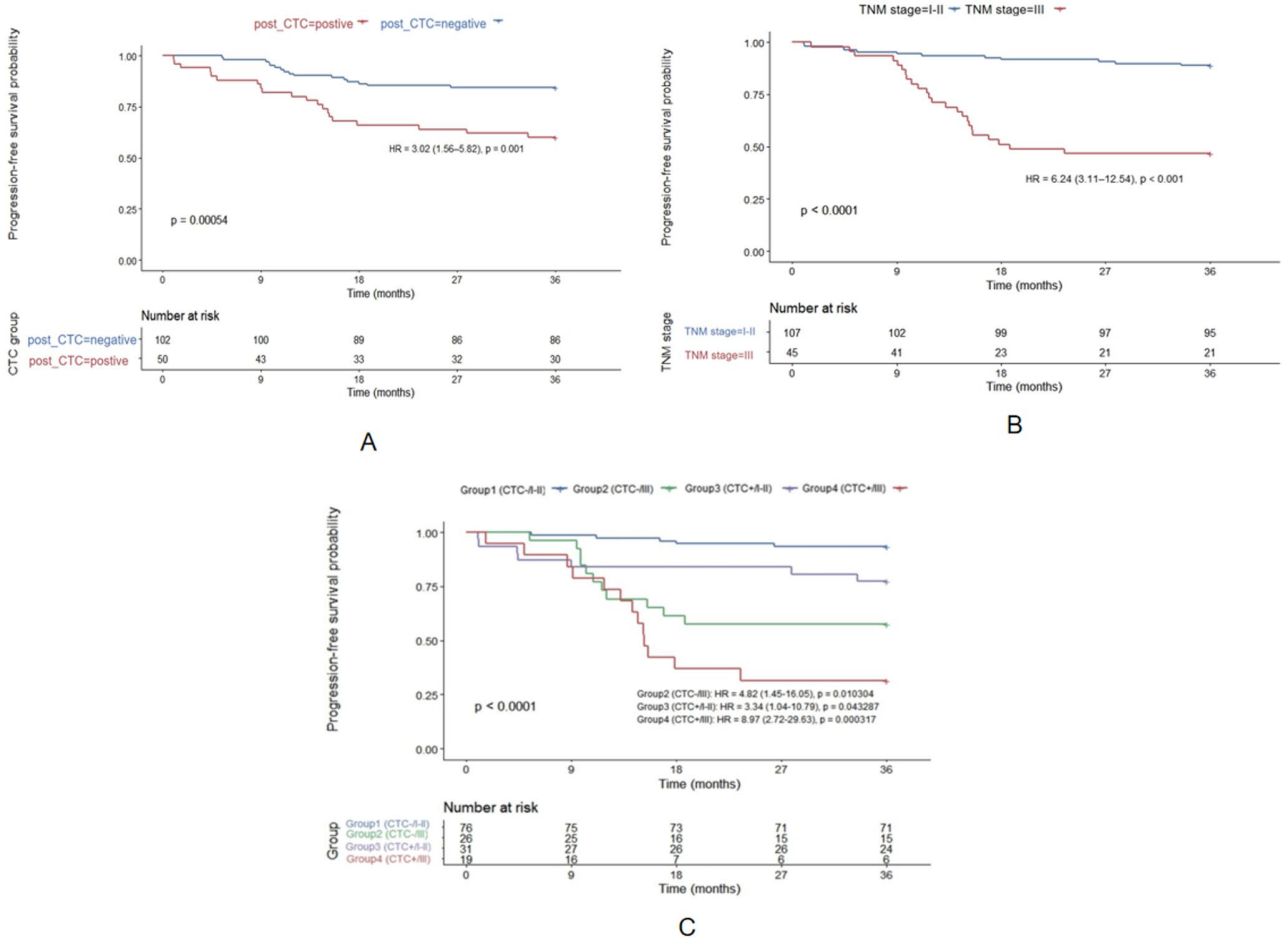
Kaplan-Meier curve displaying RFS for PD of NSCLC patients within 36 months postoperative A: Kaplan-Meier curve stratified by postopertative FR^+^CTC. B: Kaplan-Meier curve stratified by TNM stage. C: Kaplan-Meier curve stratified by TNM stage and postopertative FR^+^CTC

Stratification by postoperative FR^+^CTC status revealed that FR^+^CTC[+] patients exhibited a shorter PFS than FR^+^CTC[-] patients (26.6 months vs. 32.4 months, *P* = 0.005). Patients with postoperative FR^+^CTC[+] had a 3.02-fold higher risk of PD compared to postoperative FR^+^CTC[-] patients (HR = 3.02, 95%CI: 1.56–5.82, *P* = 0.001) **(**Fig. 2A**)**.Stratified analysis by TNM stage showed patients with TNM stage III had a shorter median PFS than those with stage I–II too (23.37 months vs. 33.45 months, *P* < 0.0001), with a 6.24‑fold increased risk of PD (HR = 6.24, 95% CI: 3.11–12.54, *P* = 0.001) **(**Fig. 2B). To investigate the predictive value of combining postoperative CTC detection results with TNM staging for the risk of disease PD, we further stratified patients into the following four groups based on these two parameters: Group 1 (postoperative FR^+^CTC[-], TNM stage I-II), Group 2 (postoperative FR^+^CTC[-], TNM stage III), Group 3 (postoperative FR^+^CTC[+], TNM stage I-II), and Group 4 (postoperative FR^+^CTC[+], TNM stage III). Survival curve analysis revealed that patients in Group 4 had the highest rate of PD progression (*P* < 0.0001, Fig. 2C), suggesting that this combined stratification strategy effectively identifies a high-risk population.

### Correlation between dynamic changes of FR^+^CTC and postoperative PD within 36 months in NSCLC patients

As shown in Table 5, stratification of patients based on the dynamic changes of FR+ circulating tumor cells (FR^+^CTC) revealed a distinct gradient in the incidence of postoperative PD.Specifically, in the NN group (preoperative FR^+^CTC [-] and postoperative FR^+^CTC [-]), PD occurred in only 6 (11.76%) patients. The incidence of PD increased progressively to 10(19.61%) patients in the PN group (preoperative FR^+^CTC [+] and postoperative FR^+^CTC [-]), 15 (37.50%) patients in the PP group (preoperative FR^+^CTC [+] and postoperative FR^+^CTC [+]), and peaked at 5(50.00%) patients in the NP group (preoperative FR^+^CTC [-] and postoperative FR^+^CTC [+]). A statistically significant trend was observed across the four groups (*P* = 0.006).

**Table 5:** Correlation between dynamic changes of FR^+^CTC and postoperative PD within 36 months in NSCLC patients

	NN *n*(%)	PN *n*(%)	PP *n*(%)	NP *n*(%)	*P*
no-PD	45(88.24)	41(80.39)	25(62.50)	5(50.00)	**0.006**
PD	6(11.76)	10(19.61)	15(37.50)	5(50.00)	

NN group: preoperative FR^+^CTC [-] and postoperative FR^+^CTC [-], PN group: preoperative FR^+^CTC [+] and postoperative FR^+^CTC [-], PP group: preoperative FR^+^CTC [+] and postoperative FR^+^CTC [+], NP group: preoperative FR^+^CTC [-] and postoperative FR^+^CTC [+]. Bold values indicate statistical significance ( *P* < 0.05).

**Fig. 3:**
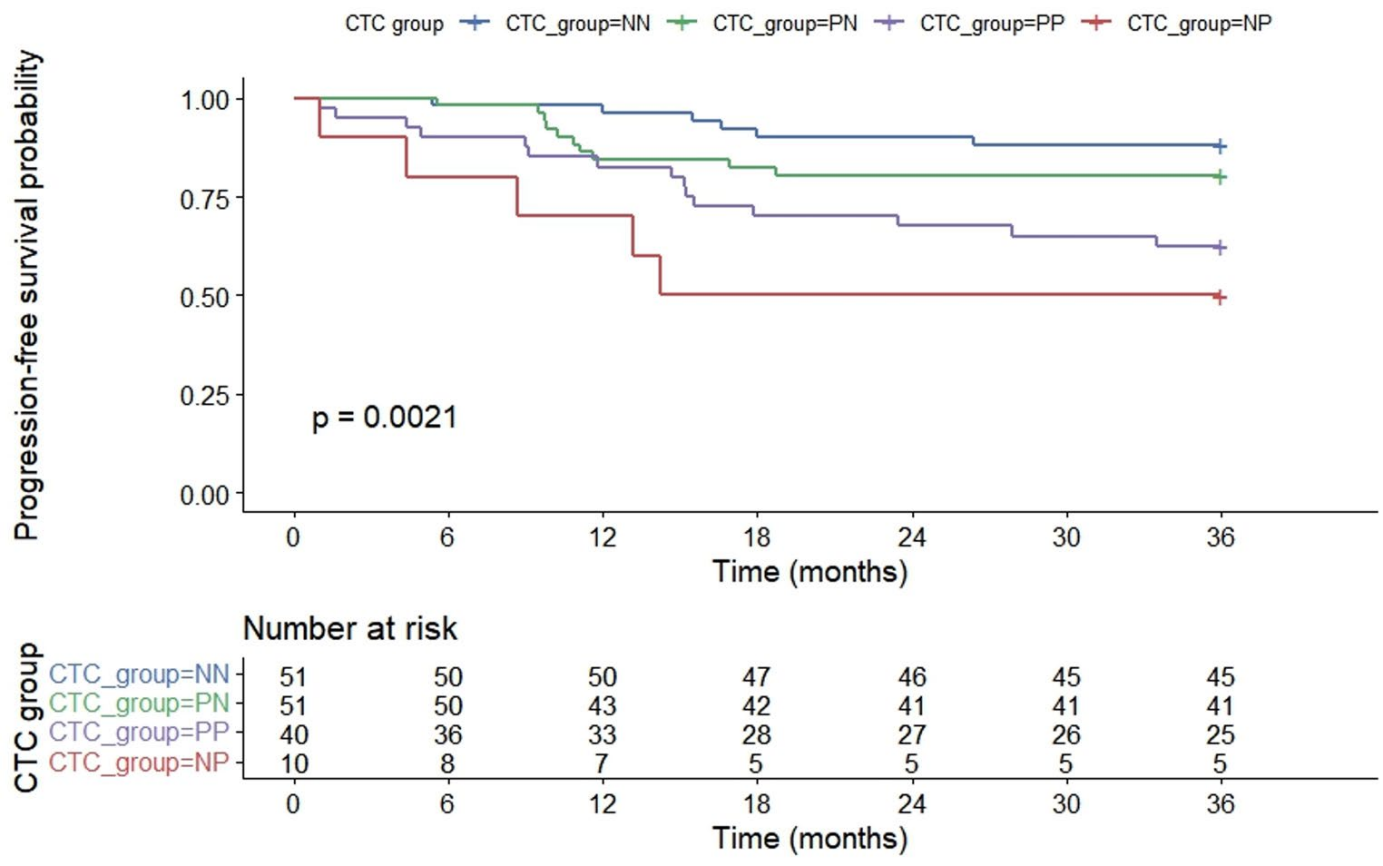
Kaplan-Meier curve stratified by preoperative FR^+^CTC and postoperative FR^+^CTC change of PFS in NSCLC patients

Kaplan–Meier curve of PD of stratified by preoperative and postoperative changes show that, A significant difference in PFS was observed among the four groups (Log-rank test, *P* < 0.05); The NP group established highest HR for PD (Fig. 3).

NN group: preoperative FR^+^CTC [-] and postoperative FR^+^CTC [-], PN group: preoperative FR^+^CTC [+] and postoperative FR^+^CTC [-], PP group: preoperative FR^+^CTC [+] and postoperative FR^+^CTC [+], NP group: preoperative FR^+^CTC [-] and postoperative FR^+^CTC [+].

## Discussion

In this study we enrolled NSCLC patients who underwent radical resection surgrey, detected preoperative FR^+^CTC and preoperative FR^+^CTC, explored the risk factors for PD within 36 months and the value of FR^+^CTC as a risk predictive molecular biomarker.

The preoperative detection rate of FR^+^ CTC in NSCLC patients was 57.30% in our study, which is lower than that reported in previous studies utilizing similar techniques (Xu et al 2023, Qin et al 2021). This discrepancy likely stems from our cohort’s distinct composition, characterized by a higher proportion of stage I–II patients and the exclusion of those with stage IIIB–IV disease. Regarding preoperative status, CTC positivity was significantly associated with male gender and Stage III. In the postoperative setting, significantly higher rates of FR^+^ CTC were observed in smokers, patients with SCC, and those with Stage III disease.The observed correlation between male sex, smoke, stage III disease, and FR^+^CTC[+]—a finding consistent with prior literature—likely reflects the more aggressive tumor biology inherent to these clinical profiles. Such factors are known to facilitate tumor cell detachment and intravasation, thereby increasing the likelihood of CTC detection in the peripheral blood (Wang et al. 2024, Kimbrough et al. 2024, Jang et al. 2024, Yang et al. 2023).The elevated postoperative FR + CTC positivity rate observed in patients with SCC may be attributed to the distinct biological behavior of this histological subtype. While conventional wisdom suggests that adenocarcinoma has a greater predilection for hematogenous metastasis, our findings—consistent with those of Xu et al. (Xu et al. 2023)—highlight that persistent postoperative CTCs in SCC patients also warrant significant clinical attention.

Follow-up data revealed that 23.02% of patients developed PD within 36 months postoperatively. Univariate analysis demonstrated that male sex, smoke, SCC histology, stage III disease, and postoperative FR^+^CTC[+] were significantly associated with an increased risk of PD. These findings are consistent with previously reported high-risk clinical features linked to postoperative recurrence and metastasis (Kimbrough et al. 2024, Jang et al. 2024, Ouyang et al. 2025),indicating that patients with these clinical characteristics may warrant more rigorous postoperative monitoring and follow-up. However, adjuvant therapy was also identified as a significant risk factor for postoperative PD in the univariate analysis, which is inconsistent with clinical expectations. This counterintuitive finding is most likely due to confounding by indication: patients with higher tumor burden, more advanced TNM stage, or poorer pathological differentiation—who inherently have a higher propensity for disease progression and recurrence—are more likely to receive routine adjuvant therapy, not because adjuvant therapy itself promotes tumor progression. Of particular note, the postoperative FR^+^CTC positivity rate was significantly higher in the PD group than in the non-PD group, whereas no significant difference was observed in the preoperative FR^+^CTC positivity rate. This finding suggests that postoperative CTC detection may better reflect MRD and metastatic potential compared with preoperative detection. Subsequent multivariate Cox regression analysis for risk factors of disease progression demonstrated that postoperative FR^+^CTC[+] and stage III disease were independent predictors of PD within 36 months after surgery in patients with NSCLC. Patients with postoperative FR^+^CTC[+] exhibited a 2.25‑fold higher risk of PD compared with those who were FR^+^CTC[-]. These results are consistent with those reported by Ma et al. (Ma et al. 2024), who demonstrated a 1.9‑fold increased risk of recurrence in patients with high levels of FR^+^CTC relative to those with low levels.

A recent meta-analysis by Wang et al. (Wang et al. 2024) encompassing 22 studies and 1674 NSCLC patients provided robust evidence supporting that patients with persistent or post-treatment CTC positivity exhibited significantly inferior PFS and overall survival (OS) compared to those with CTC clearance (HR = 1.71 for PFS, HR = 1.50 for OS).The prognostic model incorporating gender, smoking status, histological subtype, TNM stage, and postoperative FR^+^CTC status achieved good discriminatory power for postoperative PD within 36 months in NSCLC patients, with a C-index of 0.80. Its predictive performance was comparable to conventional TNM staging and previously reported clinicopathology-derived prognostic models (Kim et al. 2025).The PFS analysis also demonstrated that the combination of postoperative FR^+^CTC status and TNM stage exhibits greater incremental clinical value for PD risk prediction, indicating a markedly elevated risk of postoperative PD within 36 months.

Our study also identified a significant association between dynamic changes in FR^+^CTC status and PD. Patients with preoperative FR⁺CTC negativity who subsequently turned FR⁺CTC positive after surgery (the NP group) presented a PD rate as high as 50.00%, which was markedly higher than that of other subgroups, including the persistently FR⁺CTC-positive group.A similar study by Akikazu Kawase et al. (Kawase et al. 2025), which enrolled 29 Japanese patients with stage I NSCLC, also demonstrated that immediate postoperative detection of CTCs, particularly in those with preoperative CTC negativity, serves as an independent risk factor for tumor recurrence and is significantly associated with vascular invasion and distant metastasis.

Consistent with previous reports (Wang et al. 2024, Kawase et al. 2025), our findings further support that the persistence of postoperative FR⁺CTC likely reflects undetectable micrometastatic residual disease and the inherent metastatic potential of circulating tumor cells. Of note, the inferior prognosis of patients with FR⁺CTC conversion (negative-to-positive) can be reasonably explained by two potential mechanisms. First, surgical trauma and intraoperative tumor manipulation can trigger the shedding of dormant tumor cells into the peripheral circulation, which may convert patients from preoperative CTC-negative to postoperative CTC-positive status, further elevating the risk of early distant recurrence and disease progression (Tohme et al 2017, Kawaguchi et al 2024). Alternatively, such postoperative FR⁺CTC positivity may serve as an early indicator of MRD reactivation or inherent treatment resistance, implying that these tumors possess more aggressive biological phenotypes and poorer sensitivity to routine adjuvant therapy (Javed et al 2023, Zhang et al 2024).

Our findings demonstrate that dynamic monitoring of perioperative FR⁺CTC changes offers excellent predictive value for 36-month postoperative PD in NSCLC patients. Patients with FR⁺CTC negative-to-positive conversion warrant intensified clinical surveillance, earlier imaging follow-up, consideration of intensified adjuvant therapy strategies, and prioritized enrollment in interventional clinical trials.

However, several limitations of the present study should be acknowledged. First, this was a single-center retrospective study with a relatively limited sample size, which may introduce potential selection bias. Second, molecular and genomic information, including EGFR, ALK and KRAS status, was not available in our cohort. Accordingly, the impact of molecular subtypes on postoperative disease progression could not be further stratified and evaluated. Third, FR⁺CTC were measured only once preoperatively and once postoperatively; this single time-point design cannot dynamically reflect the longitudinal changes of FR⁺CTC after surgery. Further large-sample, multicenter prospective studies are warranted to validate the predictive value of FR⁺CTC and optimize postoperative monitoring strategies for NSCLC patients after radical resection.

## Conclusions

In summary, our study identified postoperative FR⁺CTC positivity and TNM stage III as independent risk factors for 36‑month PD following radical resection in patients with NSCLC. Postoperative FR⁺CTC[+] was associated with significantly shorter PFS and served as an effective predictor of postoperative PD. Patients with a shift from preoperative FR⁺CTC negativity to postoperative FR⁺CTC positivity carried the highest risk of disease progression. Both postoperative FR⁺CTC and dynamic monitoring of preoperative and postoperative FR⁺CTC changes demonstrated potential as liquid biopsy biomarkers for predicting PD risk within 36 months in patients with NSCLC after radical surgery.

## Data Availability

All raw data generated and analyzed in this study are available from the corresponding author on reasonable request.
